# Barriers to early and effective overactive bladder management in male patients with lower urinary tract symptoms

**DOI:** 10.1371/journal.pone.0328723

**Published:** 2025-07-23

**Authors:** Paul Rasner, Maria Arcila-Ruiz, José Ailton Fernandes Silva, Umaphorn Nuanthaisong, Sung Yung Cho, Eric Chung

**Affiliations:** 1 Urological Department, Moscow State University of Medicine and Dentistry, Moscow, Russia; 2 Department of Urology, Hospital Universitario San Ignacio, Bogota, Colombia; 3 Department of Urology, Pedro Ernesto University Hospital-State University of Rio de Janeiro, Rio de Janeiro, Brazil; 4 Faculty of Medicine, Vajira Hospital, Navamindradhiraj University, Bangkok, Thailand; 5 Department of Urology, Seoul National University Hospital-Seoul National University College of Medicine, Seoul, South Korea; 6 Department of Urology, Princess Alexandra Hospital, The University of Queensland, Brisbane, Australia; Jan Biziel University Hospital No 2 in Bydgoszcz: Szpital Uniwersytecki Nr 2 im dr Jana Biziela w Bydgoszczy, POLAND

## Abstract

Overactive bladder is underdiagnosed and undertreated in men as symptoms overlap with benign prostatic obstruction. This study identified barriers urologists encountered in effectively managing overactive bladder in men with lower urinary tract symptoms. 60 urologists recruited across Australia, Brazil, Colombia, Russia, South Korea and Thailand via convenience sampling participated in in-depth interviews. They were asked about their understanding and management of overactive bladder in men with lower urinary tract symptoms. Thematic analysis identified six themes: (1) overactive bladder diagnosis in men with lower urinary tract symptoms is complicated by the multi-factorial causes of storage symptoms; (2) patient-reported outcome tools are underutilized by urologists; (3) stigmatization and normalization of overactive bladder discourage men from seeking care; (4) challenges exist in tracking overactive bladder symptoms and in patient-urologist communication; (5) the underestimation of overactive bladder contributes to the non-urgency of its management; and (6) urologists may not fully appreciate the trade-offs between different overactive bladder treatments. Encouraging patient-reported outcome tool usage and differentiating the safety profiles of antimuscarinics and beta-3 agonists may facilitate timelier overactive bladder diagnosis and increase confidence in adding-on treatment.

## Introduction

Overactive bladder (OAB) is a symptom defined by urinary urgency, usually accompanied by increased daytime frequency and/or nocturia, with or without urinary incontinence, in the absence of urinary tract infection or other detectable disease, such as metabolic disorders or other pathologies [1,2]. OAB is a subset of the broader category known as lower urinary tract symptoms (LUTS), which comprises symptoms related to the lower urinary tract originating from the bladder, urethra, prostate and/or adjacent pelvic floor or pelvic organs, or from similarly innervated anatomy [3]. OAB can co-exist with other LUTS.

The prevalence of OAB varies across studies [4]. The EPIC study is the largest international population-based cross-sectional survey on urinary incontinence, OAB and other LUTS to date, conducted among 19,165 individuals in Canada, Germany, Italy, Sweden, and the United Kingdom. It reported the prevalence of OAB to be 11.8%, similar between men and women [5]. However, other studies reported lower prevalence among men (10% – 20%) than women (33%) [6]. OAB impairs quality of life (QoL) and productivity, disrupting daily activities such as sleep, social and sexual functions. It is also associated with higher rates of depression and stress [4,7]. QoL is most compromised among patients experiencing urinary incontinence, and its degree of impairment increases with symptom severity. Therefore, reducing OAB symptom severity can improve QoL [4].

However, OAB is underdiagnosed and undertreated [6]. The EPIC study found that only 60% of respondents with OAB symptoms sought medical care and 27% received treatment [7]. Similar trends are observed in Asia-Pacific and Latin America. A cross-sectional study involving participants from China, Taiwan, and South Korea found that less than half (47%) of men with OAB sought medical attention for their urinary symptoms [8]. In Brazil, a study found that just 35.1% of men with OAB sought treatment [9]. The diagnosis of OAB is made based on symptoms alone [10], in the absence of urinary tract infection or other detectable disease (previously termed primary OAB) [1,2,11]. While other diseases or pathologies can also contribute to storage symptoms, they may not fully explain the symptoms. In such cases, OAB becomes an additional diagnosis that co-exists with those pathologies (previously termed secondary OAB). This overlap and complexity make it difficult to identify and diagnose OAB correctly.

Particularly in men, OAB symptoms may be incorrectly attributed to benign prostatic obstruction (BPO), as symptoms of both conditions may overlap [7]. While the EPIC study found OAB prevalence to be similar between men and women, treatment was disproportionate – only one male patient was treated for every four female patients [7]. This suggests that OAB management in male patients may face more complexities than in female patients. As LUTS in men is traditionally attributed to BPO [7], male LUTS patients generally receive α-blockers as initial treatment, as per clinical recommendations [12,13]. Patients with an increased risk of disease progression, for example, if their prostate volume exceeds 40 milliliters, may additionally be prescribed 5-α-reductase inhibitors (5αRIs) [13]. However, these agents alone are often insufficient in improving OAB-related storage symptoms in male patients [7,12].

To address storage symptoms that persist despite α-blocker monotherapy, the 2022 European Association of Urology (EAU) guidelines for non-neurogenic male LUTS recommend adding-on antimuscarinics or beta-3 agonists [13]. Similarly, the American Urological Association (AUA) strongly recommends clinicians to offer antimuscarinics or beta-3 agonists to patients with OAB to improve urinary urgency, frequency, and/or urinary incontinence [14]. Antimuscarinics are the mainstay of OAB pharmacological treatment, but are associated with side effects such as urinary retention, dry mouth and constipation [7,13,15]. Real-world data suggests that side effects are key contributors of low antimuscarinic treatment persistence among OAB patients [6,15]. Urologists may also hesitate to add-on antimuscarinics due to urinary retention concerns [7], although evidence for this remains mixed – some studies suggest an increased risk of urinary retention with antimuscarinics [35], while others do not [33]. Conversely, beta-3 agonists demonstrate comparable efficacy to antimuscarinics while exhibiting greater tolerability [13,15]. This has been shown to translate into greater treatment persistence [13].

Given the prevailing gaps in OAB care among men with LUTS, it is imperative to understand the barriers urologists face in managing these patients. While there are existing studies that investigate the prevalence of OAB, there is a notable lack of research that explores the barriers to effective OAB management in men with LUTS from the perspective of healthcare providers.

Therefore, the objective of this study is to identify common barriers that urologists in different countries encounter in effectively managing OAB, particularly in male LUTS patients. Findings from this study serve as a foundational step to enhance the standard of care for OAB in men with LUTS.

## Materials and methods

### Study design

This was a cross-sectional, qualitative research study comprising in-depth interviews among urologists across six countries from different parts of the world. Using a multi-national approach enabled the identification of key barriers that urologists face across diverse healthcare and cultural settings. Australia, South Korea and Thailand were selected to represent the Asia-Pacific perspective, while Brazil and Colombia were selected to represent a Latin American perspective. Russia was also included for a Russian perspective. In determining the geographic scope of this study, regions with a scarcity of literature on OAB diagnosis and treatment barriers were prioritized. Within these regions, countries were selected to ensure variability in the cultural contexts and health systems represented. The identification of common barriers across these countries, despite their varied contexts, would suggest that they are widely experienced and are, therefore, key barriers to OAB management in men with LUTS. Where notable differences between countries emerge, these were acknowledged as areas necessitating further investigation.

Development of the interview discussion guide was informed by a literature review and the Capability, Opportunity, Motivation and Behavior (COM-B) framework, an evidence-based behavioral change model [16]. Questions in the discussion guide addressed each component of the COM-B model to systematically and comprehensively capture individual-level factors that influenced urologists’ behavior and practices in managing OAB in male LUTS patients (Table 1 and S1).

**Table 1:** Discussion guide questions mapped to COM-B components.

COM-B component	COM-B component in the context of this study	Example of relevant discussion guide questions
Capability—physical	Skill, ability or proficiency acquired through practice and experience in managing OAB in men with LUTS	How do you determine whether the root cause of LUTS in your male patients is due to BPO or OAB?
Capability—psychological	Knowledge and awareness of OAB in men with LUTS, its characteristics, impact and treatment options	Please explain why you would prescribe each of these treatments, in terms of efficacy
Opportunity—physical	Environmental opportunities such as time and resources that influence their management of OAB in men with LUTS	How are these outcomes measured? E.g., what tools do you use to measure them?
Opportunity—social	Interpersonal processes that influence one’s thoughts, feelings or management of OAB in men with LUTS	To what extent do you think these patients are communicating the full impact of their condition with you?
Motivation—automatic	Involuntary processes such as desires, impulses and inhibitions in the context of OAB in men with LUTS	Please explain why you would prescribe each of these treatments, in terms of treatment familiarity
Motivation—reflective	Contemplative processes such as evaluating the consequences of an action on OAB in men with LUTS	Please expand on why you agree/disagree that “it is important to identify and treat OAB in male patients with LUTS as quickly as possible”

BPO, Benign Prostatic Obstruction; COM-B, Capability, Opportunity, Motivation, Behavior; LUTS, Lower Urinary Tract Symptoms; OAB, Overactive Bladder.

Given the multi-national scope of the study, the discussion guide, which was developed in English, was translated into local language where applicable – Portuguese for Brazil, Spanish for Colombia, Russian for Russia, Korean for South Korea, and Thai for Thailand. The original English language discussion guide was used for Australia. Two cognitive interviews per country were conducted to optimize and validate the discussion guide in local languages.

### Sampling and data collection

Recruitment and data collection were conducted between 10 July 2023 and 5 August 2023. In-depth interviews were conducted among a total of 60 urologists, comprising 10 urologists from each country. The urologists were recruited via convenience sampling against a set of screening criteria and were approached by email or telephone. Urologists responsible for prescribing pharmacological treatment to a minimum number of male LUTS patients with OAB were recruited. Urologists who strongly recommended surgery over pharmacological treatment were excluded. Efforts were made to recruit an even representation of urologists from public and private practice. The sample size was determined before data collection and was designed to attain data saturation. Past research suggests that 9–17 interviews sufficiently achieved data saturation in qualitative research [17].

The interviews lasted approximately 60 minutes each and were conducted either face-to-face or via teleconference without others present. All interviews were moderated by experienced qualitative interviewers fluent in the local language (S2 Table). While the co-authors developed the interview discussion guides, they did not conduct the interviews. The urologists interviewed did not have any prior relationships with the co-authors or interviewers. No repeat interviews were carried out and all interviews were audio-recorded for transcription purposes only. Neither transcripts nor study findings were shared with interviewees.

The respondents were asked about their understanding and current practices towards OAB in male LUTS. This included OAB’s disease burden in terms of symptoms, QoL impact, diagnosis, and initiation of OAB add-on atop BPO treatment. Treatment monitoring, adjustment and patients’ treatment persistence were also explored. Urologists’ unmet needs in clinical practice were investigated to uncover barriers to optimal patient management. The open-ended nature of the discussion guide questions allowed participants to speak of their clinical experience freely and spontaneously. Each interview was transcribed, and where applicable translated, for data analysis in English.

### Data analysis

Data analysis was conducted by two data coders using Microsoft Word. Both inductive and deductive thematic analysis approaches were used. Responses gathered from the interviews were first analyzed via an inductive approach where transcripts were coded into conceptual categories. Each transcript was first read line-by-line in its entirety to build data familiarity. Thereafter, preliminary coding was conducted where excerpts which contributed to answering the research question were identified and assigned initial descriptive labels. The codes were then grouped to form themes and subthemes through repeated comparisons. Using a deductive approach, themes and subthemes were mapped to the COM-B framework. Mapping the themes to urologists’ capability, opportunity and/or motivation factors will help to inform behavioral change strategies to promote effective management of OAB in men with LUTS. As themes began to reoccur, it was deemed that data saturation had been attained. The final set of themes was reviewed, refined, and defined into core themes. As a quality assurance step, an additional researcher independently reviewed 10% of the transcripts to verify the replicability and reliability of data analysis. The research team members, consisting of urology experts based in the selected countries, ultimately concurred with the core themes in a final discussion meeting, affirming its consistency with their experience and observations in clinical practice.

### Ethics and quality statement

The study was granted exemption from ongoing regulatory requirements and Institutional Review Board (IRB) oversight by Pearl IRB (IN, US, IRB ID: 2023−0219), according to 45 CFR 46.104(d)(2) Tests, Surveys, Interviews. The study was conducted according to the principles of the Declaration of Helsinki. All respondents provided written informed consent for participation after being informed of the interview objectives, the voluntary nature of their participation and a guarantee of their confidentiality and anonymity. The COREQ (COnsolidated criteria for REporting Qualitative research) Checklist was adhered to and is provided as S3 Table.

## Results

A total of 60 urologists were interviewed. Apart from South Korea (where hospitals are categorized differently), 26% of urologists practiced in public hospitals, 38% in private hospitals, and 36% practiced in both (Table 2). In South Korea, 60% and 40% practiced in tertiary and general hospitals respectively. Overall, most urologists had five to 19 years of experience in practice and saw a monthly average of 20–99 male LUTS patients with OAB.

**Table 2:** Participant characteristics.

	Urologists (N = 60)
**Countries**	
Australia	**10**
Brazil	**10**
Colombia	**10**
Russia	**10**
South Korea	**10**
Thailand	**10**
**Practice (excluding South Korea)**	
Public	**13** (26.0%)
Private	**19** (38.0%)
Mixed	**18** (36.0%)
**Practice (South Korea)**	
Tertiary hospital	**6** (60.0%)
General hospital	**4** (40.0%)
**Years of practice**	
<5	**5** (8.3%)
5–9	**19** (31.7%)
10–14	**15** (25.0%)
15–19	**14** (23.3%)
≥20	**7** (11.7%)
**Monthly caseload of male LUTS patients with OAB**	
<20	**10** (16.7%)
20–49	**12** (20.0%)
50–99	**19** (31.7%)
100–199	**9** (15.0%)
≥200	**10** (16.7%)

LUTS, Lower Urinary Tract Symptoms; OAB, Overactive Bladder.

### Themes identified from interviews

The following major themes were identified: (1) OAB diagnosis in men with LUTS is complicated by the multi-factorial causes of storage symptoms; (2) patient-reported outcome (PRO) tools are underutilized by urologists; (3) stigmatization and normalization of OAB discourage men from seeking care; (4) challenges exist in tracking OAB symptoms and in patient-urologist communication; (5) the underestimation of OAB contributes to the non-urgency of its management; and (6) urologists may not fully appreciate the trade-offs between different OAB treatments. Narrative excerpts and citations are provided throughout the section and in S4 Table to illustrate participants’ perspectives. Each theme is mapped to its relevant COM-B component from a urologist’s perspective, as presented in Table 3.

**Table 3:** Themes mapped to COM-B components.

Theme	COM-B component(s)
OAB diagnosis in men with LUTS is complicated by the multi-factorial causes of storage symptoms	– Capability (physical, psychological)
Patient-reported outcome (PRO) tools are underutilized by urologists	– Opportunity (physical, social) – Motivation (reflective)
Stigmatization and normalization of OAB discourage men from seeking care	– Opportunity (social)
Challenges exist in tracking OAB symptoms and in patient-urologist communication	– Capability (physical) – Opportunity (social)
The underestimation of OAB contributes to the non-urgency of its management	– Motivation (automatic, reflective)
Urologists may not fully appreciate the trade-offs between different OAB treatments	– Capability (psychological)

LUTS, Lower Urinary Tract Symptoms; OAB, Overactive Bladder.

### OAB diagnosis in men with LUTS is complicated by the multi-factorial causes of storage symptoms

Many urologists intuitively associate male LUTS with BPO and OAB with female patients (see S4 Table, Q1). Therefore, at first consultation, urologists may not consider OAB as a potential contributor to storage symptoms in male patients.

“There is an incorrect impression of OAB being gendered in that it’s a female disease. And so, I think they see a man walk in the door and if he’s got bladder symptoms, [they] immediately [focus on] the prostate.” (P19)

Additionally, the multi-factorial causes of storage symptoms make it difficult to pinpoint OAB as a cause. Comorbidities (e.g., diabetes, BPO) (Q2 – Q4), lifestyle factors (e.g., caffeine consumption) (Q5, Q6) and aging can all contribute to storage symptoms. Moreover, urologists mentioned that their male patients with both BPO and OAB tended to be older (>50 years) and more comorbid than BPO-only patients (<50 years).

“To confirm OAB in our patients, often we have to rule out many other conditions. So if we rule out all possible conditions, but symptoms still persist, we can confirm OAB.” (P54)

### PRO tools are underutilized by many urologists

Less than half of urologists reported using PRO tools, such as the International Prostate Symptom Score (IPSS), Overactive Bladder Symptom Score (OABSS) and bladder diaries, to guide OAB management. Of urologists using PRO tools, most were observed to be from Russia and South Korea and were more likely to make treatment adjustments within a month of initiation. Urologists’ rationale for PRO use included enabling baseline comparisons of symptoms (Q7), identification of the need to adjust treatment (Q8) and challenging patients’ verbal reporting should they contradict (Q9).

“If the size of the prostate is less than 50g, and… if the score is 8 or higher in the OABSS questionnaire… tamsulosin + mirabegron is prescribed.” (P25)

Urologists who recognized the value of PRO tools proactively helped patients access and complete them effectively through printouts (Q10), digital applications (Q11), and selecting simple, patient-friendly PRO tools such as the OABSS (Q12).

Nevertheless, the majority of urologists interviewed mentioned that treatment decisions are ultimately centered around patients’ verbal reports and satisfaction. Urologists often only consider the diagnosis of OAB when patients complain of persistent storage symptoms despite BPO treatment. This is because OAB is generally not perceived as life-threatening, but rather, a non-urgent condition that primarily impacts QoL (Q13). Therefore, OAB management is observed to only begin from follow-up consultations, up to three months away. Even if OAB is identified, urologists may delay adding-on treatment, should patients claim to be satisfied despite persistent storage symptoms (Q14, Q15).

“OAB never killed anybody… So, [the decision to add-on OAB treatment] depends on how much [OAB] bothers the patients, how much it affects their QoL.” (P31)

Urologists who rely on patients’ verbal reports perceived existing PRO tools as unnecessary, time-consuming, and not patient-friendly (Q16 – Q20). This trend was more pronounced in countries other than Russia and South Korea, and was consistently seen among urologists in both public and private practice, regardless of years of experience or seniority levels.

“I do not do IPSS because that table seems very long, cumbersome, it requires academic preparation for the patient who reads it and we handle patients of all sociocultural levels.” (P42)

Nonetheless, a few of these urologists acknowledged the usefulness of PRO tools in understanding patient experiences and symptomatic improvements. As a compromise, some reportedly administered selected questions from PRO tools (Q21, Q22).

“I try to summarize the IPSS because it is difficult to reach the patient [and for them] to understand.” (P45)

### Stigmatization and normalization of OAB discourage men from seeking care

Urologists acknowledged that men feel stigmatized and ashamed of their storage symptoms, contributing to reluctance in seeking medical care (Q23). Additionally, men may normalize OAB symptoms as part of ageing (Q24).

“Some feel ashamed because…social embarrassment. The smell of the urine, need to use diapers.” (P34)

“Patients…consider that it is normal to have such problems in their age.” (P56)

Urologists reported that their male patients typically seek care only when forced to, either by their family (Q25) or when symptoms become intolerable. However, even if they do seek care, they may downplay their OAB experience (Q26). Sensitive topics like sexual (e.g., erectile dysfunction) and mental health (e.g., anxiety and depression) are not often fully discussed between patients and urologists (Q27, Q28).

“I think the poor mental health is the biggest concern that gets underrated and underplayed… Quite a few numbers of patients are depressed because of their condition. And I think we don’t really delve much into it.” (P15)

### Challenges exist in tracking OAB symptoms and in patient-urologist communication

Even when men seek medical care, urologists note their struggles in understanding, identifying and tracking the correct symptoms. Urgency and nocturia were symptoms identified as overly complex for patients to comprehend and accurately track (Q29 – Q31).

“Some patients may consider [night urination] to be lying awake and getting up to urinate at night... However, that’s not the definition of night urination for urologists as the patient does not wake up due to the need to urinate.” (P3)

Should patients successfully track their symptoms, some urologists noted that patients face yet another barrier in accurately articulating their symptoms to urologists (Q32, Q33).

In the absence of PRO tools, the onus is on urologists to ask the right questions to extract the necessary information from patients (Q34). This is challenging, as patients may be dishonest, for example, when reporting treatment compliance (Q35, Q36).

“My experience is [that] patients will lie to you. They’re very unreliable.” (P12)

Additionally, many urologists find probing time-consuming and challenging as their communication skills and willingness to engage patients vary (Q37 – Q39). These communication barriers limit urologists’ understanding of patients’ OAB experience (Q26, Q40).

### The underestimation of OAB contributes to the non-urgency of its management

The aforementioned barriers contribute to OAB in men with LUTS being underdiagnosed, and its prevalence underestimated by urologists (Q41).

“We tend to underestimate the incidence of [OAB in men with LUTS]… We see the reports in the literature. And they are much higher than what we end up seeing in practice. We end up believing that’s the way it is.” (P37)

Nonetheless, urologists were optimistic that diagnosis is improving, as current medical training considers OAB in men with LUTS more, compared to its previous prostate focus (Q42). However, urologists still report greater use of the IPSS over OABSS, and BPO remains the focus of treatment over OAB (Q43, Q44).

“Well, I see it in my own colleagues. I see it in patients who have been sent for a second opinion, who I think have bleedingly obvious diagnosis of OAB, and they’ve been offered a transurethral resection of the prostate.” (P19)

Only a third of urologists appreciated the urgency of diagnosing and treating OAB early, citing implications such as worsening symptomatology (Q45), kidney dysfunction (Q46) and psychogenic neurological issues (Q47). OAB’s QoL impact is also underestimated. When presented with evidence of OAB’s impact on sexual health, more than a third of urologists saw it as new information. Even when sexual health impact was acknowledged, it was typically attributed to treatment side effects (e.g., retrograde ejaculation from dutasteride) rather than OAB itself (Q48). Some urologists spontaneously mentioned that QoL is less important in elderly patients, who are assumed to be retired, less active, and thus less bothered by daily inconveniences (Q49).

“[OAB] doesn’t create a lot of burden in terms of impact on physical health because older people usually accept the physical changes, they are not afraid, they have poorer mental health, they’re usually retired, and they don’t have much of an economic burden…” (P3)

With an incomplete understanding of OAB’s disease burden, the perceived drawbacks of adding-on OAB treatment outweigh the benefits. These drawbacks include polypharmacy (Q50), added treatment cost and potential side effects (Q51) which can lead to treatment non-persistence. Moreover, adding-on OAB treatment risks compromising BPO treatment (Q52).

“The problem with OAB medication is that they are bladder suppressants… if the patient takes OAB medication alongside LUTS medication, the patient may struggle to urinate.” (P3)

Therefore, urologists may delay adding on OAB treatment to allow more time for BPO treatment to take “full effect” (Q53), underscoring their non-urgency in diagnosing and treating OAB.

“The medication used to treat LUTS often also improves OAB symptoms to some extent. That is, OAB can sometimes wait.” (P1)

### Urologists may not fully appreciate the trade-offs between different OAB treatments

When asked to describe their OAB treatment approach among male LUTS patients, most urologists described a similar approach. Patients who first present with obstructive outflow tract symptoms or an enlarged prostate are typically prescribed α-blockers (with/without 5αRIs). However, the time between treatment initiation and re-evaluation varied widely from 3 weeks to 3 months. At follow-up, about 10% of patients would report improvements in their obstructive symptoms but persistent OAB symptoms. These patients would be prescribed OAB add-on treatment. Across urologists, beta-3 agonists and antimuscarinics were prescribed at comparable frequencies.

Most urologists (57%) perceived beta-3 agonists and antimuscarinics as equally efficacious. 25% perceived beta-3 agonists as more effective in reducing the frequency of storage symptoms, and 18% felt that antimuscarinics were more effective, especially at higher doses.

In terms of safety profile, most urologists recognized beta-3 agonists as safer; and associated with fewer side effects such as dry mouth and constipation (Q54). Beta-3 agonists are therefore preferred over antimuscarinics for elderly patients (Q55) and those with comorbidities (e.g., cognitive disorders, constipation, glaucoma, autoimmune diseases).

“[Beta-3 agonists are] probably the best, because it doesn’t have constipation, dry mouth.” (P13)

However, beyond dry mouth and constipation, beta-3 agonists’ reduced risk of urinary retention compared to antimuscarinics did not resonate with most urologists (Q56, Q57) (only 20% spontaneously mentioned). Urologists who acknowledged beta-3 agonists’ reduced risk of urinary retention suggested that prescribing it can increase patients’ acceptance of OAB add-on treatment (Q58).

“Intuitively, people worry about going into retention with the OAB drugs, and we’ve got good literature which says it doesn’t happen. And I think if this was out there, people would be inclined to treat OAB symptoms early.” (P12)

Recognizing beta-3 agonists’ full safety profile also helped urologists in tailoring their treatment approach based on the key concerns of each patient, such as urinary retention (Q59, Q60).

“Since [beta-3 agonists have] fewer side effects related to residual urine after voiding compared to anticholinergic drugs, this can be relatively safely used in male LUTS patients who are anxious that they suddenly may not be able to urinate anytime and anywhere.” (P24)

Treatment costs were reported as one of several barriers to prescribing beta-3 agonists. Almost a third of urologists mentioned reserving beta-3 agonists for more affluent patients. Less affluent patients were perceived as more willing to accept antimuscarinic side effects (Q61, Q62). Additionally, some urologists mentioned that they or their colleagues tend to prescribe antimuscarinics habitually, sometimes without considering alternatives (Q63).

“Some doctors are so used to prescribing [antimuscarinics], they do not consider any other options.” (P56)

## Discussion

This is the first multi-national qualitative study that uncovers the barriers to OAB diagnosis and optimal treatment among male LUTS patients from urologists’ perspectives. Key diagnosis barriers were identified to be the multi-factorial causes of storage symptoms, underutilization of PRO tools, stigmatization of OAB, normalization of its symptoms, and limitations in patient-urologist conversations. As a result of underdiagnosis, urologists underestimate the impact of OAB in men and lack the urgency to initiate treatment. When OAB treatment is initiated, if at all, treatment may be suboptimal as urologists may not fully appreciate the differentiating benefits and drawbacks of treatment options.

Urologists acknowledged that diagnosing OAB is inherently complex due to its overlapping symptoms with other LUTS such as BPO and stress incontinence [18]. Storage symptoms may also be a result of other physical and cognitive disabilities, underlying comorbidities and environmental factors [6]. These multifactorial causes may overshadow the possibility of bladder-related issues, especially in men where the focus often defaults to the prostate. As a result, the possibility of OAB, with or without BPO, only begins to be considered when storage symptoms persist despite BPO treatment. While BPO is more prevalent than OAB, approximately 40% of men with BPO also have OAB [19]. Considering the possibility of OAB only from follow-up consultations can therefore delay diagnosis and appropriate OAB care for a considerable patient population for extended periods [20].

Urologists who took a more proactive approach to identifying OAB reported usage of PRO tools such as the IPSS, OABSS and bladder diaries to enable baseline comparisons or validate patients’ verbal accounts. This practice was observed more frequently among Russian and South Korean urologists, warranting further exploration of inter-country differences in clinical practice. Future studies should validate the association between PRO tool usage and timelier OAB treatment adjustments. The use of PRO tools has been recommended by several international guidelines, including the AUA and the EAU, to facilitate OAB diagnosis and evaluate treatment success [13,21]. However, we found that PRO tools were used only by a minority of urologists or were used on an infrequent basis. Similar to past reports, low PRO tool utilization stemmed from urologists’ perception that they are impractical and time-consuming [22–24]. This may stem from the logistical need to carry writing material, the time needed to transpose data collected, calculate results, and log them into patients’ medical records [22]. Perceptions of PRO tools being time-consuming in nature may stem from urologists’ greater use of the IPSS, which comprises seven lengthy prostate-focused questions [25]. However, studies have demonstrated the IPSS to be inadequate in diagnosing OAB [25]. Conversely, the OABSS is a validated self-assessment tool comprising four simple OAB-focused questions [25]. Urologists’ adapted use of the IPSS (i.e., administering select questions) may imply receptivity towards more practical and efficient tools like the OABSS.

Relying on patients to initiate complaints of persistent storage symptoms will inevitably miss patients who do not complain due to a higher tolerance of storage symptoms [26]. In turn, these patients do not obtain the necessary treatment, limiting opportunities to improve their QoL [26]. Moreover, urologists note that patients may be reluctant to speak of their storage symptoms due to embarrassment, contributing to underreporting [23,27]. Patients may also normalize their storage symptoms, believing that they are a result of comorbidities or ageing and therefore do not warrant medical attention [23]. Even if patients choose to seek medical help, they may face difficulties articulating their symptoms accurately and urologists may not effectively lead patients through conversations about OAB. Hahn et al. (2017) found that physician-patient communication about OAB was physician-centric rather than patient-centric [28]. Physicians often dominated the conversation and asked closed-ended questions without inviting patients to share their OAB experience. This resulted in misaligned understanding, especially regarding the impact of OAB on QoL and adherence to treatment [28]. Inadequate communication between patients and urologists will inevitably limit the diagnosis and treatment of OAB [23].

**Fig 1:**
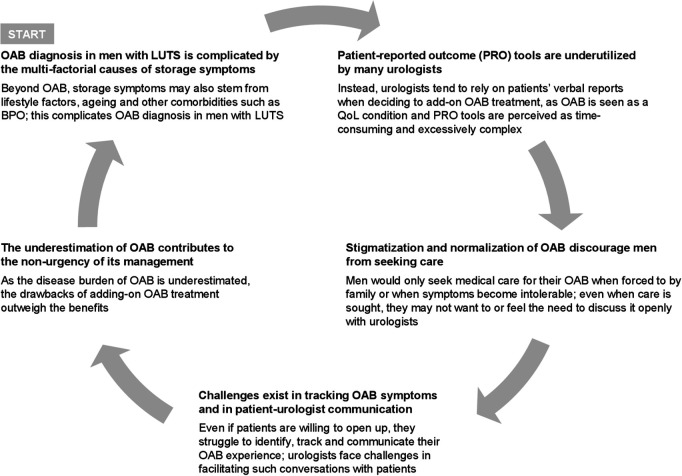
Cycle of OAB underdiagnosis in men with LUTS. BPO, Benign Prostatic Obstruction; LUTS, Lower Urinary Tract Symptoms; OAB, Overactive Bladder; QoL, Quality of Life; PRO, Patient-Reported Outcome.

These barriers to OAB diagnosis suggest a cycle that reinforces the underdiagnosis and underestimation of its impact (Fig 1). Similar cycles have been observed in various medical conditions, most notably regarding mental health [29]. Cycles of neglect and invisibility have also been documented for chronic respiratory disease, perpetuating underdiagnosis and creating barriers to care and treatment [30]. In the context of OAB, underdiagnosis reinforces its hidden nature where it continues to be overshadowed by the other multi-factorial causes of storage symptoms. As underdiagnosis creates a false perception that OAB is of low prevalence, urologists may not proactively identify OAB. This in turn feeds back into this cycle.

Delayed or underdiagnosis of OAB also results in an underestimation of its impact. If the full impact of OAB is not realized, urologists may be hesitant to add on OAB treatment as they perceive its drawbacks such as potential side effects, cost and polypharmacy to outweigh the benefits [6,15,31].

Where OAB treatment is prescribed, urologists in our study prescribed antimuscarinics and beta-3 agonists at comparable frequencies. Multiple studies, including a meta-analysis and pooled clinical trial data analysis, have demonstrated that beta-3 agonists have a lower risk of side effects compared to antimuscarinics [13,15,32]. Most urologists recognized beta-3 agonists as the safer alternative with a lower risk of side effects such as dry mouth and constipation. However, only a minority of urologists acknowledged beta-3 agonists’ reduced risk of urinary retention as compared to antimuscarinics, despite citing urinary retention as a concern when deciding to add-on OAB treatment. Existing evidence of the risk of urinary retention with antimuscarinic use is mixed. A 2011 systematic review reported the incidence of acute urinary retention in men receiving antimuscarinics with or without α-blockers to be ≤ 3% [33]. Nevertheless, the authors of the review still recommend performing a post-void residual assessment before antimuscarinic treatment and monitoring for urinary retention post-antimuscarinic treatment for men with increased risk of urinary retention [33], such as those of older age, with prostate enlargement, more severe LUTS, or are taking higher doses of antimuscarinics [33,34]. On the other hand, a more recent systematic review and meta-analysis published in 2023 found that the risk of urinary retention, increased residual urine volume and/or dysuria significantly increased with antimuscarinics use (relative risk [RR]: 2.88, P < 0.001) but not beta-3 agonists (RR: 1.26, P = 0.708) [35]. It is noted that while more urinary retention cases were observed with antimuscarinic use, very few patients required catheterization [35]. The risk of urinary retention with antimuscarinic use is also recognized in international guidelines, with the AUA recommending that antimuscarinics should be used with extreme caution in some OAB patients such as those with a history of urinary retention [14], and the EAU recommending that antimuscarinics should not be used in men with a post-void residual volume > 150 millilitres [13]. The EAU guidelines also acknowledge that the higher tolerability of beta-3 agonists has been shown to bring about tangible patient benefits, including greater treatment persistence, in turn leading to indirect QoL improvements [13].

These identified barriers to OAB diagnosis and treatment result in missed or suboptimal treatment of OAB in men with LUTS which worsens symptoms [20]. Several behavioral change strategies can be implemented in response, as a step towards improving the standard of care of OAB in male LUTS patients. Raising awareness among urologists of the underestimated prevalence and impact of OAB can prompt them to actively consider OAB, alongside BPO. This can be facilitated through regional or international scientific meetings aimed at strengthening clinician education. In clinical practice, this can also be actualized by administering the OABSS from the first consultation to monitor OAB symptoms systematically and facilitate timely treatment add-on. Urologists should be encouraged to initiate patient discussions on sensitive topics such as sexual and mental health to uncover the full extent of OAB’s QoL impact and initiate timely OAB treatment add-on. To facilitate such discussions, the value of appropriate PRO tools in guiding diagnostic and treatment decisions should be highlighted to urologists. Flexible administration methods, such as online versions, can also be offered to encourage consistent usage. At the same time, public awareness initiatives and patient education can raise awareness and destigmatize OAB, so patients do not dismiss or normalize their storage symptoms. Patients can also be informed of the potential long-term complications associated with unresolved storage symptoms to encourage treatment-seeking behaviors. Beyond timely OAB identification and treatment add-on, it is imperative that urologists optimize OAB treatment. Urologists should be familiarized with the QoL and safety benefits of beta-3 agonists to increase prescription confidence and patient acceptance of OAB treatment add-on.

There are several limitations to this study. Firstly, the study findings may not be generalizable due to the exploratory nature of the study. As the first multi-national study on urologists’ barriers to effective OAB diagnosis and treatment, this research aims to lay the groundwork for future investigations, including potential subgroup analyses of the current dataset. The participant eligibility criteria selected for qualified urologists relevant to our research question and convenience sampling was adopted for recruitment. This means that findings can only be interpreted directionally and may not be representative of all urologists in each country. Future studies should consider larger and more representative study populations in each country to garner more robust insights, from which implementation strategies can be tailored. Additionally, the study is not designed for inter-country comparisons. Country-specific nuances identified should therefore be validated with future studies.

## Conclusions

Urologists reported barriers in diagnosing OAB promptly and optimizing treatment for their male LUTS patients in clinical practice. The multi-factorial causes of storage symptoms, stigmatization and normalization of OAB, and suboptimal patient-urologist communication reinforce a cycle of OAB underdiagnosis. Urologists and patients require support to ensure that timelier OAB diagnosis and add-on treatment initiation can be achieved. Enabling a greater appreciation of the safety and QoL benefits of beta-3 agonists can also drive timelier OAB treatment add-on and improve persistence.

## Supporting information

S1 Table Screener and discussion guide mapped to the Capability, Opportunity, Motivation and Behavior (COM-B) framework. X = Mapped. BOO, Bladder Outlet Obstruction; BPO, Benign Prostatic Obstruction; KOLs, Key Opinion Leaders; LATAM, Latin America; LUTS, Lower Urinary Tract Symptoms; OAB, Overactive Bladder; QoL, Quality of Life.
(DOCX)

S2 Table Interviewer details. MBA, Master of Business Administration.
(DOCX)

S3 Table COREQ checklist. (PDF)

S4 Table Supporting quotes from urologist interviews. BPO, Benign Prostatic Obstruction; IPSS, International Prostate Symptom Score; LUTS, Lower Urinary Tract Symptoms; OAB, Overactive Bladder; OABSS, Overactive Bladder Symptom Score; QoL, Quality of Life; PRO, Patient-Reported Outcome.
(DOCX)
