# Correction: Barriers to early and effective overactive bladder management in male patients with lower urinary tract symptoms

**DOI:** 10.1371/journal.pone.0335425

**Published:** 2025-10-24

**Authors:** Paul Rasner, Maria Arcila-Ruiz, José Ailton Fernandes Silva, Umaphorn Nuanthaisong, Sung Yong Cho, Eric Chung

The fifth author’s name is spelled incorrectly. The correct name is: Sung Yong Cho. The correct citation is: Rasner P, Arcila-Ruiz M, Silva JAF, Nuanthaisong U, Cho SY, Chung E (2025) Barriers to early and effective overactive bladder management in male patients with lower urinary tract symptoms. PLoS One 20(7): e0328723. https://doi.org/10.1371/journal.pone.0328723.
