# Supplementary material for: Barriers to early and effective overactive bladder management in male patients with lower urinary tract symptoms
Source: PLoS One. 2025 Jul 23;20(7):e0328723. doi: 10.1371/journal.pone.0328723 (PMC12286356; doi:10.1371/journal.pone.0328723)
Supplement: S1 Table — X = Mapped. BOO, Bladder Outlet Obstruction; BPO, Benign Prostatic Obstruction; KOLs, Key Opinion Leaders; LATAM, Latin America; LUTS, Lower Urinary Tract Symptoms; OAB, Overactive Bladder; QoL, Quality of Life. (DOCX) [file pone.0328723.s001.docx]

**S1 Table.** Screener and Discussion Guide mapped to the Capability, Opportunity, Motivation and Behavior (COM-B) framework

| **No.** | | | **Key questions** | | | | **Capability (physical)** | **Capability (psychological)** | **Opportunity (social)** | **Opportunity (physical)** | **Motivation (automatic)** | **Motivation (reflective)** |
| --- | --- | --- | --- | --- | --- | --- | --- | --- | --- | --- | --- | --- |
| Screener: *Identifying eligible candidates to be interviewed* | | | | | | | | | | | | |
| 1.1 |  |  | Specialty  *Note: Must be a urologist* | | | |  |  |  |  |  |  |
| 1.2 |  |  | Seniority  *Note: At least associate or consultant* | | | |  |  |  |  |  |  |
| 1.3 |  |  | Which of the following statements best describes your treatment approach towards male patients with LUTS? | | | |  |  |  |  |  |  |
|  | 1.31 |  |  | *I will always initiate pharmacological treatment and only reserve surgery as a last resort* | | |  |  |  |  |  | x |
|  | 1.32 |  |  | *I find myself recommending an even mix of pharmacological treatment and surgery across all my male LUTS patients* | | |  |  |  |  |  | x |
|  | 1.33 |  |  | *I strongly recommend surgery instead of pharmacological treatment for all my eligible male LUTS patients*  *Note: Screen out if respondents identify with this statement i.e. surgical focus* | | |  |  |  |  |  | x |
| 1.4 |  |  | Personally responsible for prescribing pharmacological treatment / drugs to manage OAB in male patients with LUTS | | | |  |  |  |  |  |  |
| 1.5 |  |  | Thinking about your male OAB patients with LUTS, approximately how many might you manage with pharmacological treatment / drugs in an average month? | | | |  |  |  |  |  |  |
| 1.6 |  |  | Please describe how frequently you prescribe each of these treatments for male OAB patients with LUTS on a scale of 1 to 7 with 1 being "I have never prescribed it", 4 being "I prescribe on occasion" and 7 being "I prescribe it all the time". | | | |  |  |  | x |  |  |
|  | 1.61 |  |  | Alpha blocker | | |  |  |  | x |  |  |
|  | 1.62 |  |  | Antimuscarinics | | |  |  |  | x |  |  |
|  | 1.63 |  |  | Beta-3 adrenergic receptor agonist | | |  |  |  | x |  |  |
| 1.7 |  |  | Attitudes and experiences towards OAB in male patients with LUTS | | | |  |  |  |  |  |  |
|  | 1.71 |  |  | Agreement with the following statements on a scale of 1 to 7 with 1 being strongly disagree and 7 being strongly agree | | |  |  |  |  |  |  |
|  |  | 1.711 |  |  | *I am comfortable identifying OAB in male patients with LUTS* | |  | x |  |  |  |  |
|  |  | 1.712 |  |  | *It is important to identify and treat OAB in male patients with LUTS as quickly as possible* | |  |  |  |  |  | x |
|  |  | 1.713 |  |  | *Alpha blocker monotherapy provides adequate symptom control of OAB in most male patients with LUTS* | |  |  |  |  |  | x |
| Warm up: *Explore patient profile and attitude towards OAB in male LUTS to set the context for subsequent discussion on treatment, disease burden and unmet needs* | | | | | | | | | | | | |
| 2.1 |  |  | How do your male OAB patients with LUTS differ from other male LUTS patients without OAB? | | | | x |  | x |  |  | x |
|  | 2.11 |  |  | Demographic profile | | | x |  |  |  |  |  |
|  | 2.12 |  |  | Socioeconomic status | | | x |  |  |  |  |  |
|  | 2.13 |  |  | Symptoms | | |  |  |  |  |  | x |
|  | 2.14 |  |  | QoL before and after treatment | | |  |  |  |  |  | x |
|  | 2.15 |  |  | Comorbidities | | |  |  |  |  |  | x |
|  | 2.16 |  |  | Patient activation / level of engagement in managing their condition | | |  |  | x |  |  |  |
| 2.2 |  |  | **Refer to 1.5:** How, if at all, do you see the numbers of male OAB patients with LUTS changing in the next 3 years? Why? | | | |  |  |  |  |  | x |
| 2.3 |  |  | How do you determine whether the root cause of LUTS in your male patients is due to BPO or OAB? | | | | x | x |  |  |  |  |
|  | 2.31 |  |  | **Refer to 1.711:** To what extent does your approach influence your rating here? What else did you factor into your rating here? | | |  | x |  |  |  |  |
| 2.4 |  |  | How much of a priority is treating male OAB patients with LUTS vs. all the other conditions that you manage and treat (e.g. BPO)? Why? | | | |  |  |  |  |  | x |
|  | 2.41 |  |  | Unique challenges of diagnosing, managing and treating male OAB patients with LUTS vs. the other conditions you see (e.g BPO)? Why? | | |  | x |  |  |  | x |
| 2.5 |  |  | **Refer to 1.712:** Please expand on why you agree/disagree that *"it is important to identify and treat OAB in male patients with LUTS as quickly as possible"* | | | |  |  |  |  |  | x |
| 2.6 |  |  | In cases where you’ve established OAB as the cause of a male patients’ LUTS, what impact would you say their condition has on their day to day activities and quality of life generally? Why? | | | |  |  |  |  |  | x |
|  | 2.61 |  |  | Sleep | | |  |  |  |  |  | x |
|  | 2.62 |  |  | Work / productivity | | |  |  |  |  |  | x |
|  | 2.63 |  |  | Daily routine | | |  |  |  |  |  | x |
|  | 2.64 |  |  | Social life | | |  |  |  |  |  | x |
|  | 2.65 |  |  | Physical activities | | |  |  |  |  |  | x |
|  | 2.66 |  |  | Mental health | | |  |  |  |  |  | x |
|  | 2.67 |  |  | Sexual activity | | |  |  |  |  |  | x |
|  | 2.68 |  |  | Others | | |  |  |  |  |  | x |
| 2.7 |  |  | What are the clinical symptoms that your male OAB patients with LUTS find the most troubling? Why? | | | |  |  | x |  |  |  |
|  | 2.71 |  |  | To what extent do you think these patients are communicating the full impact of their condition with you? Why / why not? | | |  |  | x |  |  | x |
| Treatment approaches for OAB in male LUTS: *Map treatment algorithm and understand perception of beta-3 agonist add-on* | | | | | | | | | | | | |
| 3.1 |  |  | To what extent is your treatment approach for male OAB with LUTS (including any decision not to prescribe pharmacological treatment) influenced by guidelines or your colleagues or KOLs? | | | |  |  | x | x |  |  |
|  | 3.11 |  |  | What do these guidelines state / recommend? Are these always followed? Why / why not? | | |  |  |  | x |  |  |
|  | 3.12 |  |  | Which colleagues / KOLs influence your approach? Why? | | |  |  | x |  |  |  |
| 3.2 |  |  | Thinking about when you first discuss treatment options with your male OAB patients with LUTS, what typically happens? | | | |  |  |  |  | x |  |
|  | 3.21 |  |  | What do you typically say to / tell these patients? | | |  |  |  |  | x |  |
|  | 3.22 |  |  | How do you present treatment options? Do you typically present one option only, or go through multiple options? Why / Why not? | | |  |  |  |  | x |  |
|  | 3.23 |  |  | What questions do patients tend to have for you at this point? | | |  |  | x |  |  |  |
|  | 3.24 |  |  | How do you address their questions? | | |  |  | x |  |  |  |
| 3.3 |  |  | Thinking about pharmacological treatment for your male OAB with LUTS patients, what option would you typically start with? | | | |  |  |  |  |  | x |
|  | 3.31 |  |  | Please provide a % breakdown of pharmacological treatments prescribed | | |  |  |  |  |  | x |
|  | 3.32 |  |  | Please explain why you would prescribe each of these treatments, in terms of | | |  | x | x | x | x | x |
|  |  | 3.321 |  |  | | Efficacy |  | x |  |  |  |  |
|  |  | 3.322 |  |  | | Safety - are you or your patients particularly concerned about any side effects? Why these? |  | x | x |  |  |  |
|  |  | 3.323 |  |  | | Patients' ability to afford treatment (dependent on treatment cost or insurance coverage) |  |  | x |  |  | x |
|  |  | 3.324 |  |  | | Guidelines or departmental norms |  |  | x | x |  |  |
|  |  | 3.325 |  |  | | Patient preference |  |  | x |  |  |  |
|  |  | 3.326 |  |  | | Treatment familiarity |  |  |  |  | x |  |
|  |  | 3.327 |  |  | | How is the eventual treatment option decided upon? Who has the final say? |  | x | x |  |  | x |
| 3.4 |  |  | For each treatment option, what are the expected treatment outcomes? To what extent does this vary patient to patient? | | | |  |  |  |  |  | x |
|  | 3.41 |  |  | **If not mentioned, ask:** How if at all would each treatment impact the following: | | |  |  |  |  |  | x |
|  |  | 3.411 |  |  | | Symptoms |  |  |  |  |  | x |
|  |  | 3.412 |  |  | | Sleep |  |  |  |  |  | x |
|  |  | 3.413 |  |  | | Work / productivity |  |  |  |  |  | x |
|  |  | 3.414 |  |  | | Daily routine |  |  |  |  |  | x |
|  |  | 3.415 |  |  | | Social life |  |  |  |  |  | x |
|  |  | 3.416 |  |  | | Physical activities |  |  |  |  |  | x |
|  |  | 3.417 |  |  | | Mental health |  |  |  |  |  | x |
|  |  | 3.418 |  |  | | Sexual activity |  |  |  |  |  | x |
|  |  | 3.419 |  |  | | Others |  |  |  |  |  | x |
|  | 3.42 |  |  | How are these outcomes measured? | | |  |  |  | x |  |  |
|  | 3.43 |  |  | Which are most important / impactful to you as a clinician? Why? What does this allow you to do? | | |  |  |  |  |  | x |
|  | 3.44 |  |  | Which are most important / impactful to patients? Why? What does this allow them to do? | | |  |  | x |  |  | x |
| 3.5 |  |  | At what point do you follow up with patients? | | | |  |  |  |  | x |  |
|  | 3.51 |  |  | What happens at the follow up appointment? | | |  |  |  |  | x |  |
|  | 3.52 |  |  | What do you discuss with patients? | | |  |  | x |  | x |  |
|  | 3.53 |  |  | **PROBE**: Symptom frequency / severity / type? Treatment-related side effects or issues? | | |  |  |  |  |  | x |
| 3.6 |  |  | How do you determine if treatment is successful? | | | |  |  |  | x |  |  |
|  | 3.61 |  |  | **PROBE**: interviewing patients on symptom changes, any lab tests required etc | | |  |  | x |  |  |  |
|  | 3.62 |  |  | What triggers treatment adjustment (i.e. switch or add-on)? | | |  |  | x | x |  | x |
|  |  | 3.621 |  |  | | **PROBE**: Any criteria outlined in guidelines? Lack of treatment success? Patient feedback/request? |  |  |  |  |  |  |
|  | 3.63 |  |  | For how long would the typical male OAB patient with LUTS stay on their initial treatment before an adjustment/add-on therapy is needed? | | |  |  |  |  | x | x |
|  |  | 3.631 |  |  | | **PROBE:** Under what circumstances would treatment be adjusted or added earlier than the norm? |  |  |  |  |  | x |
|  |  | 3.632 |  |  | | **PROBE:** Under what circumstances would treatment be adjusted or added later than the norm? |  |  |  |  |  | x |
| 3.7 |  |  | Approximately what proportion of male OAB patients with LUTS on their initial treatment would require a treatment adjustment (% switch vs % add-on)? | | | |  |  |  |  |  | x |
|  | 3.701 |  |  | When would you switch vs add-on to the initial treatment for your male OAB patients with LUTS? | | |  |  |  |  |  | x |
|  | 3.702 |  |  | What are the pros and cons of switching vs adding on to the initial treatment at this stage? | | |  |  |  |  |  | x |
|  | 3.703 |  |  | % breakdown across switched/added pharmacological treatments (beta-3 adrenergic receptor agonist; Dutasteride; Tildalafil; LATAM only - antimuscarinic and alpha-blocker fixed‐dose combination) | | |  |  |  |  |  | x |
|  | 3.704 |  |  | Please explain why you would switch/add each of these treatments , in terms of | | | x | x | x | x | x | x |
|  |  | 3.7041 |  |  | | Efficacy, including symptoms unresolved by initial / previous treatment |  | x |  |  |  |  |
|  |  | 3.7042 |  |  | | Safety - are you or your patients particularly concerned about any side effects? Why these? |  | x | x |  |  |  |
|  |  | 3.7043 |  |  | | Patients' ability to afford treatment (dependent on treatment cost or insurance coverage) |  |  | x |  |  | x |
|  |  | 3.7044 |  |  | | Guidelines or departmental norms |  |  | x | x |  |  |
|  |  | 3.7045 |  |  | | Patient resistance |  | x |  |  |  |  |
|  |  | 3.7046 |  |  | | Patient preference |  |  | x |  |  |  |
|  |  | 3.7047 |  |  | | Treatment familiarity |  |  |  |  | x |  |
|  |  | 3.7048 |  |  | | How is the eventual treatment decided upon? Who has the final say? | x | x | x |  |  | x |
|  | 3.713 |  | For each treatment option, what are the expected treatment outcomes? To what extent does this vary patient to patient? | | | |  |  |  |  |  | x |
|  |  | 3.714 |  | **If not mentioned, ask:** How if at all would each treatment impact the following: | | |  |  |  |  |  | x |
|  |  | 3.715 |  |  | | Symptoms |  |  |  |  |  | x |
|  |  | 3.716 |  |  | | Sleep |  |  |  |  |  | x |
|  |  | 3.717 |  |  | | Work / productivity |  |  |  |  |  | x |
|  |  | 3.718 |  |  | | Daily routine |  |  |  |  |  | x |
|  |  | 3.719 |  |  | | Social life |  |  |  |  |  | x |
|  |  | 3.72 |  |  | | Physical activities |  |  |  |  |  | x |
|  |  | 3.721 |  |  | | Mental health |  |  |  |  |  | x |
|  |  | 3.722 |  |  | | Sexual activity |  |  |  |  |  | x |
|  |  | 3.723 |  |  | | Others |  |  |  |  |  | x |
| 3.8 |  |  | **If risk of urinary retention is mentioned in 3.7042, ask:** How does this side effect impact your patients? | | | |  |  | x |  |  | x |
|  | 3.801 |  |  | And how does this side effect impact you and your management of your patients? | | |  |  |  |  |  | x |
| 3.9 |  |  | REPEAT 3.5 for switched/added treatments | | | |  |  |  |  | x | x |
| 3.1 |  |  | **If <100% in 3.7 require treatment adjustment, ask:** To what extent are the rest of these patients well controlled on initial treatment vs dropping off/stopping treatment? | | | |  |  |  |  |  | x |
|  | 3.101 |  |  | How if at all do you follow-up with patients who drop off/stop treatment? | | |  |  |  |  | x | x |
|  | 3.102 |  |  | What if anything will trigger treatment continuation for these patients? | | |  |  |  |  |  | x |
| 3.11 |  |  | What happens next to the male OAB patients with LUTS who have had a treatment switch or add-on? | | | |  |  |  |  | x |  |
|  | 3.111 |  |  | REPEAT 3.7 - 3.11 FOR additional treatments switched or added | | |  | x | x | x | x | x |
| Unmet needs: *Uncovering unmet needs in treating and managing OAB in male LUTS* | | | | | | | | | | | | |
| 4.1 |  |  | Now thinking more generally about your male OAB patients with LUTS, what would you say are the greatest challenges with managing these patients? Why? | | | |  |  |  |  |  | x |
|  | 4.11 |  |  | Are there particular groups of these patients for whom greater level of challenges exist vs. others? Why do you say that? | | |  |  |  |  |  | x |
|  | 4.12 |  |  | What would you / your patients need to have to help address these challenges? | | |  |  | x | x |  |  |
|  |  | 4.121 |  |  | | What would this allow you to do as a physician? Why is this important? |  |  | x | x | x |  |
|  |  | 4.122 |  |  | | What would this allow your patient to do? Why is this important? |  |  | x | x |  |  |
| Disease burden and communication: Uncovering the impact of disease and how best to communicate the benefits of add-on therapy | | | | | | | | | | | | |
| 5.1 |  |  | What information sources do you use to learn about new therapies, guidelines changes? Why these? | | | |  |  | x | x | x |  |
|  | 5.11 |  |  | Which sources do you use the most? Why? | | |  |  | x | x | x |  |
|  | 5.12 |  |  | Which sources do you trust the most? Why? | | |  |  | x | x |  |  |
|  | 5.13 |  |  | How effective would you say these sources are at conveying the information you need? Why do you say that? | | |  |  | x | x |  |  |
|  | 5.14 |  |  | What, if anything, would you say is missing or could make it better? Why do you say that? | | |  |  | x | x |  |  |
| 5.2 |  |  | What information sources are available to your patients to learn about their condition and/or treatment options? | | | |  |  | x |  |  |  |
| 5.3 |  |  | To what extent are information sources able to convey what your patients need to know? | | | |  |  |  |  |  | x |
|  | 5.31 |  |  | What, if anything, would you say is missing or could make it better? Why do you say that? | | |  |  | x | x |  |  |
| 5.4 |  |  | I am now going to share a series of showcards with you, which provides data and or extracts from male OAB patients with LUTS, which we would like to get your opinions on: | | | |  | x |  |  |  | x |
|  | 5.41 |  |  | **PRESENT SHOWCARD 1 ON OAB / LUTS IMPACT ON QOL** | | |  |  |  |  |  | x |
|  | 5.42 |  |  | What are your initial reactions to this data? Why do you say that? | | |  |  |  |  |  | x |
|  |  | 5.421 |  |  | | Is there anything here that surprises you? |  |  |  |  | x | x |
|  |  | 5.422 |  |  | | Is there anything here that is new information? |  |  |  |  |  | x |
|  | 5.43 |  |  | How does this make you feel? Why? | | |  |  |  |  | x | x |
|  | 5.44 |  |  | What impact, if any, does this data / information have on the way in which you would manage or treat the male OAB patients with LUTS in the future? Why do you say that? | | |  |  |  |  |  | x |
|  |  | 5.441 |  |  | | In what ways might this change current practice? Why? |  |  |  |  |  | x |
|  | 5.45 |  |  | **PRESENT SHOWCARD 2 ON SUB-OPTIMAL TREATMENT OF OAB IN MALE LUTS** | | |  | x |  |  |  |  |
|  | 5.46 |  |  | Repeat Q5.42-5.44 | | |  | x |  |  |  |  |
| 5.5 |  |  |  | What more, if anything, would you need to see or hear in order to initiate an add on therapy for your male OAB patients with LUTS? Why do you say that? | | |  |  |  |  |  | x |
|  | 5.51 |  |  |  | | What is the greatest barrier to doing this currently? Why? |  |  |  |  |  | x |
| 5.6 |  |  |  | What more, if anything, would you need to see or hear in order to initiate an add on therapy for your male OAB patients with LUTS at an **earlier point in their treatment journey**? Why do you say that? | | |  |  |  |  |  | x |
|  | 5.61 |  |  |  | | What is the greatest barrier to doing this currently? Why? |  |  |  |  |  | x |

X = Mapped. BOO, Bladder Outlet Obstruction; BPO, Benign Prostatic Obstruction; KOLs, Key Opinion Leaders; LATAM, Latin America; LUTS, Lower Urinary Tract Symptoms; OAB, Overactive Bladder; QoL, Quality of Life

SHOWCARD 1: OAB / LUTS IMPACT ON QOL

Multiple studies have demonstrated that OAB and LUTS symptoms have a **substantial, multidimensional impact on patients** using various measures such as health-related quality of life (HRQoL), International Prostate Symptom Score-Quality of Life (IPSS-QoL) and Work Productivity and Activity Impairment (WPAI) ^1,2,3,4,5^:

- Significantly and adversely impact **physical health**^1,2,3,4,5^ **and sexual function**^1,3,5^
- **Poorer mental health than people without OAB i.e., greater levels of depression, anxiety and embarrassment/shame ^1,2,3,4,5^; difficulties with social life^1,3^; impact on sleep and sexual pleasure^1,3,5^**
- **Substantial economic burden of disease related to increased absenteeism, worry about interruption and scheduling of meetings and early retirement^1,5^**

Sources:

1. Shaw C, Gibson W. Assessing Quality-of-Life of Patients Taking Mirabegron for Overactive Bladder. Ther Clin Risk Manag. 2023;19:27-33
2. Kim SK, Kim SH. The impact of overactive bladder on health-related quality of life in Korea: based on the results of a Korean Community Health Survey. Qual Life Res. 2021 Apr;30(4):1017-1024. doi: 10.1007/s11136-020-02710-3. Epub 2020 Nov 20. PMID: 33216260.
3. Kinsey D, Pretorius S, Glover L, Alexander T. The psychological impact of overactive bladder: A systematic review. Journal of Health Psychology. 2016;21(1):69-81.
4. Bartoli S, Aguzzi G, Tarricone R. Impact on quality of life of urinary incontinence and overactive bladder: a systematic literature review. Urology. 2010 Mar;75(3):491-500. doi: 10.1016/j.urology.2009.07.1325. Epub 2009 Dec 4. PMID: 19962738.
5. Coyne KS, Sexton CC, Irwin DE, Kopp ZS, Kelleher CJ, Milsom I. The impact of overactive bladder, incontinence and other lower urinary tract symptoms on quality of life, work productivity, sexuality and emotional well-being in men and women: results from the EPIC study. BJU Int. 2008 Jun;101(11):1388-95. doi: 10.1111/j.1464-410X.2008.07601.x. PMID: 18454794.

SHOWCARD 2: SUB-OPTIMAL TREATMENT OF OAB IN MALE LUTS

There is a wealth of evidence drawing attention to the opportunities to optimize OAB treatment in male LUTS patients since 2009.

Sources:

1. Wagg A. Male Overactive Bladder: Underappreciated, Under-researched. More Please? Eur Urol. 2021 Apr;79(4):505-506. doi: 10.1016/j.eururo.2021.01.004. Epub 2021 Jan 19. PMID: 33483177.
2. Burnett AL, Walker DR, Feng Q, et al. Undertreatment of overactive bladder among men with lower urinary tract symptoms in the United States: A retrospective observational study. Neurourol Urodyn. 2020;39(5):1378-1386.
3. Millman AL, Cheung DC, Hackett C, Elterman D. Overactive bladder in men: a practical approach. Br J Gen Pract. 2018;68(671):298-299.
4. Gomelsky A, Dmochowski RR. Overactive bladder in males. Therapeutic Advances in Urology. 2009;1(4):209-221.

Review papers dating **back to 2009**^4^ noted that:

- Physicians’ **‘prostate-centered’ approach** to treating LUTS in men is contingent on the assumption that urinary frequency and urgency result solely from incomplete emptying due to partial BOO
- Unfortunately, this assumption fails to consider the role of the bladder, and failure to do so may leave a **proportion of men undertreated**
- Indeed, if men with LUTS are treated with any medications, they appear to be **predominantly treated with agents for BPO rather than agents aimed at OAB**

A decade later, review papers **in 2018 and 2020**^1,2,3^ still note that:

- Some cases of LUTS due to OAB may be **attributed incorrectly** to BPO and vice versa. However, the etiology is often not distinguished in the clinical setting and a **broader diagnosis of LUTS is given**.
- When BPO is suspected, **alpha‐blockers** are often used as a primary therapy for LUTS. However, these therapies may **fail to alleviate OAB‐induced storage symptoms.**
- Inadequate management of OAB-induced storage symptoms in patients with LUTS means that **most patients do not remain on pharmacological treatment long term**

*A controlled clinical safety study in patients with BOO did not demonstrate increased urinary retention in patients treated with mirabegron; however, mirabegron should be administered with caution to patients with clinically significant BOO

*A controlled clinical safety study in patients with BOO did not demonstrate increased urinary retention in patients treated with mirabegron; however, mirabegron should be administered with caution to patients with clinically significant BOO

3
