# Supplementary material for: Barriers to early and effective overactive bladder management in male patients with lower urinary tract symptoms
Source: PLoS One. 2025 Jul 23;20(7):e0328723. doi: 10.1371/journal.pone.0328723 (PMC12286356; doi:10.1371/journal.pone.0328723)
Supplement: S2 Table — MBA, Master of Business Administration. (DOCX) [file pone.0328723.s002.docx]

**S2 Table.** Interviewer details

| **S/N** | **Country** | **Gender** | **Credentials** | **Occupation** | **Experience** |
| --- | --- | --- | --- | --- | --- |
| 1 | Russia | Female | Master’s degree | Project manager and moderator | - Intensive training on focus group management – instruments design, moderation, insights generation, reporting - Moderated in-depth interviews in the fields of urologists, uro-oncologists, endocrinologists, etc. (non-exhaustive) |
| 2 | Brazil | Female | MBA | Moderator | - 20 years of experience in market research - Experienced in conducting group discussions, in-depth interviews, ethnographic research, interviews and online groups across various therapeutic areas |
| 3 | Mexico | Female | Diploma | Managing Director and Partner | - 20 years of experience in healthcare market research - Experienced in conducting in-depth interviews, group moderations, and online focus groups among physicians, nurses, pharmacists, patients, etc. (non-exhaustive) - Experience across various therapeutic areas including urology (erectile dysfunction, prostate cancer, overactive bladder, incontinence) |
| 4 | Korea | Female | Bachelor’s degree | Project manager and moderator | - 22 years of healthcare marketing research experience - Over 1200 in-depth interview moderation experience including patient, caregiver, consumer, physician, pharmacist, nurse, lab manager, payer |
| 5 | Thailand | Female | Master’s degree | Moderator | - 10 years of moderation experience |
| 6 | Australia | Female | Bachelor’s degree | Market Research Associate | - Over 20 years of market research experience - Specialized in medical and pharmaceutical market research work |

MBA, Master of Business Administration
