# Supplementary material for: Barriers to early and effective overactive bladder management in male patients with lower urinary tract symptoms
Source: PLoS One. 2025 Jul 23;20(7):e0328723. doi: 10.1371/journal.pone.0328723 (PMC12286356; doi:10.1371/journal.pone.0328723)
Supplement: S4 Table — BPO, Benign Prostatic Obstruction; IPSS, International Prostate Symptom Score; LUTS, Lower Urinary Tract Symptoms; OAB, Overactive Bladder; OABSS, Overactive Bladder Symptom Score; QoL, Quality of Life; PRO, Patient-Reported Outcome. (DOCX) [file pone.0328723.s004.docx]

**S4 Table.** Supporting quotes from urologist interviews

| **Theme** | **Sub-theme** | **Supporting quotes** |
| --- | --- | --- |
| **OAB diagnosis in men with LUTS is complicated by the multi-factorial causes of storage symptoms** | Many urologists intuitively associate male LUTS with BPO | Q1: *“There is an incorrect impression of OAB being gendered in that it's a female disease. And so, I think they see a man walk in the door and if he's got bladder symptoms, [they] immediately [focus on] the prostate.”* (P19) |
|  | The multi-factorial causes of storage symptoms, including BPO, make it difficult to identify OAB | Q2: *“To confirm OAB in our patients, often we have to rule out many other conditions. So if we rule out all possible conditions, but symptoms still persist, we can confirm OAB.”* (P54)  Q3: *“It is believed that continued [BPO] can lead to secondary changes in the bladder, affecting the development of OAB symptoms.”* (P23)  Q4: *"Patients who have long-standing diabetes, many end up developing LUTS, with symptoms of bladder hypersensitivity. And we end up being a little in doubt. I think this is the difficulty in the case of diabetic patients. Especially with these new hypoglycemic agents that [eliminate] glucose through the urine, they end up irritating the bladder.”* (P37)  Q5: *“One of the difficulties is working out whether the OAB symptoms are, indeed, an overactive bladder or a motor urgency type of issue versus many men who just have OAB symptoms, but largely due to sensory issues and poor bladder habit, and those sorts of things.”* (P17)  Q6: *“We also consider other stimulating factors such as the consumption of caffeine or cold foods. We observe how they respond to these stimuli. Suppose they [counter] these factors, and the symptoms get worse, we can consider that the cause is leaning towards OAB.”* (P2) |
| **Patient-reported outcome (PRO) tools are underutilized by many urologists** | PRO tools enabled baseline comparisons, identification of  the need to adjust treatment and validated patients’ verbal reports | Q7: *“We can know how much it improved by comparing the baseline QoL score with the QoL score after treatment.”* (P25)  Q8*. “If the size of the prostate is less than 50g, and patients have severe OAB symptoms of LUTS, in other words, if the score is 8 or higher in the OABSS questionnaire completed by patients, tamsulosin + mirabegron is prescribed.”* (P25)  Q9: *“I compare previous questionnaires and diaries with the ones the patient brings with him. I assess the difference. Some patients come and say that nothing helps. I checked the diary and questionnaires and see the improvement. So I demonstrate this improvement to the patient.”* (P51) |
|  | Urologists who see the value of PRO tools take steps to administer these tools effectively in practice | Q10: *“We use IPSS and urination dairies for 3 days. We provide our patients with the printed template of urination diary where they record the number of urinations, the volume, the degree of urinary urgency. It is very simple.”* (P60)  Q11: *“However, some urologists do not use questionnaires or urination diaries. Now there is an app for urination diary. It is easier for the patient to use the app instead of the paper version.”* (P60)  Q12: *“OABSS is the most commonly used tool, consisting of four items that assess the change in symptom. If the patient's symptom frequency improves, it can be considered that the patient's QoL has also improved. Patients usually don’t have much difficulty answering OABSS.”* (P23) |
|  | However, majority of urologists passively rely on patients’ complaints of storage symptoms as OAB is perceived as a non-life-threatening QoL condition | Q13: *“OAB never killed anybody, it makes patients angry. So, [the decision to add-on OAB treatment] depends on how much [OAB] bothers the patients, how much it affects their QoL.”* (P31)  Q14: *“I assess the patient's satisfaction. [If] he urinates often and has difficulty urinating, but his symptoms are slightly better than before and he is satisfied, I won't change my treatment approach as long as the patient is satisfied.”* (P9)  Q15: *“I think it depends on the impact the symptoms have on the quality of life. That's my motivation. It's not a morbid disease. It's got no long-term problems. So, if the patient said, I don’t like a medication, I'll put up with it. Well, good, I'm happy with that.”* (P12) |
|  | These urologists considered PRO tools to be unnecessary, time-consuming and not patient-friendly | Q16: *“I do not use any tools, not even symptom scores… Simple interviewing is enough. There is no need to waste time calculating scores unless we are collecting data for a research paper. In real life, it is unnecessary and time-consuming.”* (P7)  Q17: *“Patients will spend more than 30 minutes trying to answer them. Even the IPSS which is the simpler questionnaire may be difficult to answer.”* (P31)  Q18: *“What I noticed with IPSS is that patients don't know how to differentiate one question from the other. Or sometimes they cheat on their answers because they want to have the “best” answers.”* (P32)  Q19: *“For the majority of patients, it’s simply whether they are happier or less bothered. In most cases, I don’t rely too much on repeating scores or repeating tests. If a patient feels they are improved and they have gone from being bothered to being happy, then I will accept that. I don’t actually care what the difference is in their flow rate. I don’t care how much their IPSS score has improved.”* (P14)  Q20: *“I do not do IPSS because that table seems very long, cumbersome, it requires academic preparation for the patient who reads it and we handle patients of all sociocultural levels.”* (P42) |
|  | Nevertheless, some of these urologists acknowledged the usefulness of PRO tools and administered only selected questions as a compromise | Q21: *“I try to summarize the IPSS because it is difficult to reach the patient [and for them] to understand.”* (P45)  Q22: *“If they are having trouble, I’ll ask them to give me a score of bother, which is just the IPSS, which is a one to six bother score. That's often useful to get them to stop and think about how much it really is affecting them. And maybe they haven't really thought about it before.”* (P19) |
| **Stigmatization and normalization of OAB discourage men from seeking care** | Men feel ashamed of their storage symptoms and may dismiss them as a part of aging | Q23: *“Some feel ashamed because [they have] to stop many times at work to go to the bathroom, social embarrassment. The smell of the urine, need to use diapers.”* (P34)  Q24: *“Patients who are poorer consider that it is normal to have such problems in their age.”* (P56) |
|  | Therefore, they do not seek medical care unless prompted | Q25: *“Wife or other family member notices that the man wakes up 4 times at night to pee. It can happen that the patient gets used to the symptoms. If they had symptoms for a long time, it may not raise suspicion that it is a health condition that can be controlled.”* (P31) |
|  | Even when men do seek medical care, they may downplay the severity of their symptoms | Q26: *“Incontinence is one [symptom] that they find very embarrassing, and they don't want to communicate it. It's usually after you probe a bit more, and they say, yes, I've had a few accidents, and then you ask a few more questions and you realize, hang on, that's a lot more than what you told me.”* (P15)  Q27: *“People are reluctant to complain about mental problems.”* (P56)  Q28: *“I think the poor mental health is the biggest concern that gets underrated and underplayed a fair bit. And if you dwell a bit more on the quality of life, quite a few numbers of patients are depressed because of their condition. And I think we don't really delve much into it.”* (P15) |
| **Challenges exist in tracking OAB symptoms and in patient-urologist communication** | OAB symptoms may be too complex for patients to comprehend and accurately track | Q29: *“Some patients may consider [night urination] to be lying awake and getting up to urinate at night... However, that's not the definition of night urination for urologists as the patient does not wake up due to the need to urinate. [Night urination] means they can't sleep and feel annoyed because they need to urine; it's different.”* (P3)  Q30: *“Of course when I use the term ‘urgency,’ patients do not know the term. But sometimes, when asked if they have difficulty holding urine or experience sudden urgency to urinate, they might misunderstand the question.”* (P28)  Q31: “*These are symptoms that patients are unsure if they are a problem or not. Sometimes they do not know whether these symptoms are problematic.”* (P2) |
|  | Patients may also struggle to articulate their symptoms accurately | Q32: *“Patients often say, “I leak urine, and it stains my clothes” but patients often have difficulty using the correct terms when describing their symptoms. The symptom of urge urinary incontinence or urine leakage after emptying the bladder is called “dribbling” but many patients simply explain that they frequently go to urinate without accurately using these specific terms.”* (P23)  Q33*: “Patients have difficulties finding words to describe their problems.”* (P55) |
|  | While the onus is on urologists to ask the right questions to extract the necessary information from patients, this is met with challenges | Q34*: “So, I think [extracting information from patients] is a clinician’s skill, not a patient’s [responsibility].”* (P18)  Q35*: “My experience is [that] patients will lie to you. They're very unreliable.”* (P12) (in context of treatment compliance)  Q36: *“I ask patients and believe them when they say they take all the drugs I have prescribed. But I know, they lie.”* (P55)  Q37: *“Sometimes, I have to be very persistent [in] asking questions.”* (P55)  Q38: *“We would have to ask leading questions to understand better, or sometimes they might mention other unrelated symptoms.”* (P2)  Q39: “*It could be because my questions were not clear enough.”* (P28)  Q40*: “If they lie to me, I cannot know.”* (P34) |
| **The underestimation of OAB contributes to the non-urgency of its management** | While urologists are optimistic that OAB diagnosis will improve, a prostate-focused approach still prevails in practice and OAB remains underdiagnosed in men with LUTS | Q41: *“We tend to underestimate the incidence of [OAB in men with LUTS], especially here in Brazil. We see the reports in the literature. And they are much higher than what we end up seeing in practice. We end up believing that's the way it is.”* (P37)  Q42: *“OAB is an underdiagnosed condition, and the diagnosis has increased a lot lately. I would think that it will increase as people gain more knowledge and training.”* (P47)  Q43: *“It's urgent to differentiate OAB from BPO because patients spend many years treating the prostate when they should be treating the OAB. This is where the urgency is.”* (P33)  Q44: *“Well, I see it in my own colleagues. I see it in patients have been sent for a second opinion, who I think have bleedingly obvious diagnosis of OAB, and they've been offered a transurethral resection of the prostate.”* (P19) |
|  | Only a third of urologists appreciate the urgency of treating OAB in a timely manner | Q45: *“Treating [OAB] early would make a huge difference in the outcomes, because if you don't treat it early, it actually makes it worse… if you treat the outlet and not the OAB it makes the symptomatology far worse.”* (P15)  Q46: *“There is a risk of deteriorating kidney function due to increased bladder pressure.”* (P42)  Q47: *“If OAB is not identified and treated in a timely manner, some neurological and psychogenic disorders can develop. Such patients will develop sleeping disorders.”* (P53) |
|  | Additionally, the QoL impact of OAB remains underestimated and undervalued | Q48: *“I doubt that OAB has any impact on sexual function.”* (P55)  Q49: *“[OAB] doesn’t create a lot of burden in terms of impact on physical health because older people usually accept the physical changes, they are not afraid, they have poorer mental health, they’re usually retired, and they don’t have much of an economic burden… However, if a 45 year-old man has this condition, it will be a burden for him.”* (P3) |
|  | With OAB’s disease burden underestimated, the drawbacks of adding-on OAB treatment outweigh the benefits | Q50: *“The trickiest patients are the elderly who use a lot of medications as I need to convince them to use more medication for a disease that is not as “serious” as diabetes or hypertension that can kill them, and not as well-known as those other diseases.”* (P32)  Q51: *“If we tell them [an OAB] medication may make it difficult for them to urinate, but may control their symptoms better, they would refuse it. They would rather prioritize being able to urinate.”* (P6)  Q52: *“The problem with OAB medication is that they are bladder suppressants. In some patients who experience urinary retention, there will be a lot of post-void residual urine; if the patient takes OAB medication alongside LUTS medication, the patient may struggle to urinate.”* (P3)  Q53: *“The medication used to treat LUTS often also improves OAB symptoms to some extent. That is, OAB can sometimes wait.”* (P1) |
| **Urologists may not fully appreciate the trade-offs between different OAB treatments** | Urologists widely acknowledged the safer profile of beta-3 agonists | Q54: *“[Beta-3 agonists are] probably the best, because it doesn’t have constipation, dry mouth.”* (P13)  Q55: *“I love that [beta-3 agonists] has almost no adverse effects, I have done very well with it in an older population.”* (P45) |
|  | However, not many urologists recognize that beta-3 agonists minimize the risk of urinary retention associated with  antimuscarinics, forming yet another barrier to prescribing beta-3 agonist | Q56: *“Mirabegron and solifenacin can cause acute urinary retention. This is a serious complication. So I would never use beta-3-agonists and anticholinergics in the patients with urine retention exceeding 100 ml.”* (P57)  Q57: *“It is especially difficult when patients with OAB symptoms also have urinary retention. OAB requires the use of anticholinergics or mirabegron, but when these medications are taken, patients may have difficulty urinating properly. Managing such cases becomes particularly challenging for OAB patients.”* (P23) |
|  | Recognizing the full safety profile of beta-3 agonists can increase patient acceptance of OAB treatment add-on and help urologists tailor their treatment approaches based on patients’ preferences | Q58: *“Intuitively, people worry about going into retention with the OAB drugs, and we've got good literature which says it doesn't happen. And I think if this was out there, people would be inclined to treat OAB symptoms early.”* (P12)  Q59: *“Since [beta-3 agonists have] fewer side effects related to residual urine after voiding compared to anticholinergic drugs, this can be relatively safely used in male LUTS patients who are anxious that they suddenly may not be able to urinate anytime and anywhere.”* (P24)  Q60: *“Anticholinergics can make urination uncomfortable [as they cannot empty their bladder], while beta-3 agonists are known to cause less discomfort during urination. Therefore, for patients with urinary discomfort, mirabegron, a beta-3 agonist, might be prescribed in combination, while those with less urinary discomfort may be given Propiverine.”* (P23) |
|  | The higher cost of beta-3 agonist is one of several barriers to prescribe the drug | Q61: *“The key reasons to prescribe anti-muscarinic add-on therapy over mirabegron add-on for OAB in male LUTS patients: It is a matter of cost, as the anti-muscarinic group is cheaper and more reimbursable for patients”* (P2)  Q62: *“It is fantastic but expensive. If mirabegron were more affordable it would be much more widely used. mirabegron is sensational. If I could, I would prescribe mirabegron to many patients, but not all of them will be able to afford it.”* (P34) |
|  | Another barrier to prescribing beta-3 agonist is urologists' tendency to prescribe antimuscarinics out of habit | Q63: *“Some doctors are so used to prescribing [antimuscarinics], they do not consider any other options.”* (P56) |

BPO, Benign Prostatic Obstruction; IPSS, International Prostate Symptom Score; LUTS, Lower Urinary Tract Symptoms; OAB, Overactive Bladder; OABSS, Overactive Bladder Symptom Score; QoL, Quality of Life; PRO, Patient-Reported Outcome
